# Global pandemic: consulting strategies in managing dental patients

**DOI:** 10.1007/s41894-020-00082-y

**Published:** 2020-09-09

**Authors:** Melissa Loh, Declan Loh, William Y. C. Loh

**Affiliations:** 1 Wirral University Teaching Hospital, Arrowe Park Road, Upton, Wirral, CH49 5PE England, UK; 2 University of Liverpool, Cedar House, Liverpool, L69 3GE England, UK; 3 Southport and Ormskirk NHS Trust, Southport, PR8 6PN England, UK

**Keywords:** COVID-19, Pandemic, Telemedicine, Consultation, Dentistry, Surgical specialties

## Abstract

During periods of pandemic disease, the practice of Dentistry must be altered incorporating the appropriate patient management strategies to reduce the transmission of disease. This article will outline the roles of remote consultations including virtual out-patient clinic appointments, telephone conversations and secure video networking systems to provide optimum patient-centered care.

## Quick reference/description

As the timescale of a pandemic stabilises over time, new modes of care delivery must be devised. The most current pandemic to have affected the world population has been the severe acute respiratory syndrome Coronavirus 2, SARS-CoV-2.

Globally, guidelines for various specialities have been issued which offers baseline guidance on the clinical response to the pandemic.

Channels of communication outside the traditional face-to-face interviews allow remote review of patients whilst aiding the provision of optimum care and putting patients’ best interests first_._ A ‘remote consultation’ is where the patient and clinician engage in a consultation method, where both parties are not ‘face-to-face’.

## Indications

1. To reduce the risk of viral transmission between clinician and patient as both parties are not in the same physical location at the same time. This is in accordance with the government’s advice for social distancing.
2. To facilitate the consultation of patients who are unable to travel (e.g. high risk patient categories including patients over the age of seventy)..
3. To enable risk stratification, which allows clinicians to appropriately triage urgent reviews and determine if a traditional ‘face-to-face’ consultation is indicated.

## Materials/instruments

1. Telephone consultations.
2. Video consultations.
3. Virtual new patient, review and follow-up clinics.
4. Smartphone technology.

## Procedure

- Remote assessment and the management of dental conditions can be appropriately assessed by a clinician. This leads to a provision of appropriate advice.
- Clear methods of history taking of the presenting complaint can be explored and tailored advice or signposting can be sought. During the height of the pandemics, dental professionals are advised to offer patients appropriate advice and prescriptions via telephone call, whilst all face-to-face urgent care is reduced.
- Virtual clinics are an example of telephone consultations with the extension of the availability of imaging. With good quality visual settings, clinicians may be able to provide a visual examination of for example a healing wound, or new appearance of a lesion.
- Authorised patient educational material could be posted for aetiologies that require conservative management.
- Consider multi-disciplinary approaches with clinicians from various specialities.
- Mindful considerations of the boundaries of remote assessment including the lack of ability to physically palpate anatomy or presenting pathologies.
- Appropriate clinical triage of patients via virtual consultation will aid in reducing the unnecessary undue pressures on our Accident and Emergency (A&E) colleagues. Urgent presentations can be streamlined to decrease the unnecessary travel and hospital environment exposure. This communication method reduces the risk of transmission between (A&E) areas, where patients could be unnecessarily exposed to pandemic disease.
- Appropriate ‘safety netting’ of concerns. For example, a consultation with a patient who reports to dysphagia due to an oro-facial swelling must be swiftly escalated.

### Telephone consultations

The use of a telephone is arguably the most readily available vehicle for non face-to-face consultations. A summary of the telephone consultation can be provided for the patient to review their virtual appointment. Telephone conversations have a tendency to cause an overly narrowed focus on presenting symptoms so clinicians must be mindful to explore the patient’s social and relevant medical history prior to isolating their main concerns. Patients can email any concerns to a secure NHS email address. This may include attachments in the form of photographs of their concerns.

### Video consultations

NHS England and NHS Improvement have issued support and guidance for providers of video consultations. These guidelines are similar to global pandemic guidance across the world. The video consultations provide a visual display of both patient and practitioner that draws a closer parallel to the traditional face to face consultation than purely audio telephone conversations. Video consultations provide opportunity for clinicians to gain visual information and cues from patients which may not be so readily captured in telephone conversations. Unlike the readily available telephone, the logistics and set up of video consultations require dedicated space for uninterrupted consultation to take place, administrative staff input and clinical staff training. There may also be issues regarding patient access to appropriate equipment and software to accommodate video consultations.

Virtual clinics are an extension of the video consultations between a single clinician and patient. They are an alternative to traditional face-to-face clinics. Virtual clinics incorporate the multidisciplinary team (MDT). An example of the MDT in the Orthopaedic surgical speciality would include an orthopaedic surgeon or senior doctor to assess and diagnose, physiotherapist or advanced nurse practitioner (ANP) to provide therapeutic guidance and administrative staff for paperwork.

If the clinician deems the patient’s clinical situation requiring a face to face consultation, this can be arranged. Special investigations such as radiographs should be organised and ordered prior to the patient attending the appointment in person to reduce total number of visits. Remote access to these special investigations should be implemented to allow optimum efficiency of clinical time.

### Smartphone technology

A smartphone is a newer class of cellular telephone which has enhanced the way we communicate as a society. It has become an essential part of today’s communication. The emergence of fifth generation (5G) technology allows instant, stable and uninterrupted flow of information across all platforms. The smartphone is a method of providing portable and instant information for the user. This information can be stored and transferred between various media. Privacy and security issues when using smartphone technology must be considered to provide confidential patient care.

### Conclusions

- Remote consultation strategies have been rapidly implemented to reduce the rate of transmission of this virus between clinicians and patients.
- The twenty-first century has witnessed a revolutionised communicative technology based society and it is these telecommunication technologies healthcare must look to everyday practice to optimise patient management.

Mandatory continuing professional development (CPD) has been affected during the global pandemic for many individuals due to the cancellation of conferences, workshops and other events. Clinicians could use the virtual clinics and remote consultations as areas of reflection in developing good communication skills which ensure they are kept up to date with the current changes in their speciality.

Webinar, virtual learning and e-learning play important roles not only in the CPD but also maintaining the social networking among participants. Looking to the future, virtual online forums and devices have replaced and will likely continue to replace the traditional face to face conferences during and after the pandemic period. They have played important roles not only in the CPD but also maintaining the ability to social network amongst participants.

## Pitfalls and complications

- Good communication encompasses verbal and non-verbal cue. Clinicians will have to place more emphasis on their verbal techniques such as tone and content of speech when displaying empathy, or sympathy as telephone conversations do not facilitate the patient viewing the clinician. Telephone consultations must take into consideration patient disabilities such as visual or hearing impairments which may form another obstacle in conducting these consultations. Despite these inherent disadvantages, Logishetty et al. reported equal patient satisfaction between face to face and telephone consultations.
- The workload for clinicians is not necessarily reduced via remote consultations. Good documentation and record keeping must remain at the forefront of a clinicians’ consultation.
- Lack of physical examination may prove difficult in formulating an accurate clinical diagnosis. For example: performing special investigations via tactile stimulus. A secure agreed network to support the various technologies which allow for remote consultations must be established in line with the General Data Protection Regulations in the European Union guidelines (GDPR) to protect patient information.

## Further reading

1. Coronavirus» Specialty guides [Internet]. England.nhs.uk. 2020 [cited 9 May 2020]. Available from: https://www.england.nhs.uk/coronavirus/secondary-care/other-resources/specialty-guides/.
2. General Dental Council—Focus On Standards [Internet]. Standards.gdc-uk.org. 2020 [cited 8 May 2020]. Available from: https://standards.gdc-uk.org/.
3. Staying at home and away from others (social distancing) [Internet]. GOV.UK. 2020 [cited 9 May 2020]. Available from: https://www.gov.uk/government/publications/full-guidance-on-staying-at-home-and-away-from-others/full-guidance-on-staying-at-home-and-away-from-others.
4. COVID-19: guidance on social distancing and for vulnerable people [Internet]. GOV.UK. 2020 [cited 9 May 2020]. Available from: https://www.gov.uk/government/publications/covid-19-guidance-on-social-distancing-and-for-vulnerable-people.
5. Acute Dental Problems—COVID-19—SDCEP [Internet]. SDCEP. 2020 [cited 9 May 2020]. Available from: https://www.sdcep.org.uk/published-guidance/acute-dental-problems-covid-19/.
6. Car J. Telephone consultations. BMJ. 2003;326(7396):966–969.
7. Galloway C. Nonverbal communication. Theory Into Practice. 1968;7(5):172–175.
8. van Galen L, Car J. Telephone consultations. BMJ. 2018;380:1–2.
9. Logishetty K, Subramanyam S. Adopting and sustaining a Virtual Fracture Clinic model in the District Hospital setting—a quality improvement approach. BMJ Quality Improvement Reports. 2017;6(1):1–10.
10. General Dental Council—Maintain and protect patients’ information [Internet]. Standards.gdc-uk.org. 2020 [cited 9 May 2020]. Available from: https://standards.gdc-uk.org/pages/principle4/principle4.aspx
11. Iyengar K, El-Nahas W. A brief guide to telephone medical consultation. British Journal of Healthcare Management. 2020;26(4):1–3.
12. Greenhalgh T, Vijayaraghavan S, Wherton J, Shaw S, Byrne E, Campbell-Richards D et al. Virtual online consultations: advantages and limitations (VOCAL) study. BMJ Open. 2016;6(1):1–13.
13. Chih-Lin, Shuangfeng Han, Zhikun Xu, Qi Sun, Zhengang Pan. 5G: rethink mobile communications for 2020 +| Philosophical Transactions of the Royal Society A: Mathematical, Physical and Engineering Sciences [Internet]. Doi.org. 2020 [cited 10 May 2020]. Available from: https://doi.org/10.1098/rsta.2014.0432.
14. Kapıcıoğlu M. The reliability of use of WhatsApp in type 1 and type 2 pediatric supracondylar fractures. Joint Diseases and Related Surgery. 2019;30(2):149–154.
15. Kavoor A, Chakravarthy K, John T. Remote consultations in the era of COVID-19 pandemic: Preliminary experience in a regional Australian public acute mental health care setting. Asian Journal of Psychiatry. 2020;51:102,074.
16. Parisien R, Shin M, Constant M, Saltzman B, Li X, Levine W et al. Telehealth Utilization in Response to the Novel Coronavirus (COVID-19) Pandemic in Orthopaedic Surgery. Journal of the American Academy of Orthopaedic Surgeons. 2020;28(11):e487–e492.

